# Machine Vision Requires Fewer Repeat Measurements than Colorimeters for Precise Seafood Colour Measurement

**DOI:** 10.3390/foods13071110

**Published:** 2024-04-04

**Authors:** Kieren Watkins, Melindee Hastie, Minh Ha, Graham Hepworth, Robyn Warner

**Affiliations:** 1 School of Agriculture, Food and Ecosystem Sciences, The University of Melbourne, Parkville, VIC 3010, Australia; kmwatkins@student.unimelb.edu.au (K.W.); melindee.hastie@unimelb.edu.au (M.H.); minh.ha3444@gmail.com (M.H.); 2 Statistical Consulting Centre, University of Melbourne, Parkville, VIC 3010, Australia; hepworth@unimelb.edu.au

**Keywords:** salmon, rockling, prawns, fish, Nix, Minolta, technical replicate, standard error of the mean, Delta E

## Abstract

The colour of seafood flesh is often not homogenous, hence measurement of colour requires repeat measurements to obtain a representative average. The aim of this study was to determine the optimal number of repeat colour measurements required for three different devices [machine vision (digital image using camera, and computer processing); Nix Pro; Minolta CR400 colorimeter] when measuring three species of seafood (Atlantic salmon, *Salmo salar*, *n* = 8; rockling, *Genypterus tigerinus*, *n* = 8; banana prawns, *Penaeus merguiensis*, *n* = 105) for raw and cooked samples. Two methods of analysis for number of repeat measurements required were compared. Method 1 was based on minimising the standard error of the mean and Method 2 was based on minimising the difference in colour over repeat measurements. Across species, using Method 1, machine vision required an average of four repeat measurements, whereas Nix Pro and Minolta required 13 and 12, respectively. For Method 2, machine vision required an average of one repeat measurement compared to nine for Nix Pro and Minolta. Machine vision required fewer repeat measurements due to its lower residual variance: 0.51 compared to 3.2 and 2.5 for Nix Pro and Minolta, respectively. In conclusion, machine vision requires fewer repeat measurements than colorimeters to precisely measure the colour of salmon, prawns, and rockling.

## 1. Introduction

The colour of seafood is important for its acceptability in the marketplace and by consumers. Colour is an indicator of freshness, as indicated by its central role in the Quality Index Method (QIM) of seafood evaluation [1]. Colour influences consumer acceptance and hedonic appeal, which can increase consumers’ willingness to pay [2,3,4]. For example, more vivid orange salmon fillets attract higher prices [5,6] which motivates salmon aquaculturists to add pigments to feed to improve their colour [7,8]. Colour can also be an indicator of how thoroughly seafood is cooked, and can vary with the cooking method used [9,10].

The food industry often uses instrumental measurements of colour due to instrument speed and consistency, and the fact that instruments are objective compared to subjective visual methods. Colour is typically measured in three dimensions: L* for lightness, a* for a spectrum from green to red, and b* for a spectrum from blue to yellow [11]. However, measuring the colour of seafood is challenging because the surfaces of many seafood species are not uniform in colour. An example of non-uniform surface colour is raw salmon, which has lines of collagen–fat matrix between the bands of muscle, resulting in alternating darker and lighter bands, which can influence the instrumental measurement of colour and grading of the fillet [12,13].

The flesh of many seafood species has colour variation over the anatomy of the body. Whilst there is no published research on the colour of rockling (*Genypterus tigerinus*) flesh, salmon has been reported to have greater pigmentation radially toward the backbone, and longitudinally toward the midsection from the tail [14,15]. Defects caused by microbes and thermal stress can also cause inconsistent colour in Atlantic salmon (*Salmo salar*) [16]. Banana prawns (*Penaeus merguiensis*) have been known to vary in colour both between and within individuals [17]. Australian prawns, such as the banana prawn, turn from white with a green pigment to white with a red pigment on cooking [18,19]. Atlantic salmon have a pink or orange colour before becoming white and slowly browning with cooking [5,20,21]. Seafood flesh in general varies in colour within and between species, and this colour is affected by many factors. The aquaculture industry and retailers are aware of the importance of seafood colour, and both production and retail have consistent colour standards for salmon, and are working towards establishing them for other species [22,23]. Thus, robust objective methods for colour measurement are required.

Differences in colour reading instruments and their settings can also influence the measured colour values, so the same object can register as different colours due to instrumental differences. The aperture size, observer angle, and light source vary between models and brands of instruments [24,25,26,27,28,29,30,31,32,33]. The nature of the material being measured and the retail display environment determine the instrument settings, such as whether to include specular reflection and choice of illuminant, which all influence the measured colour [11,27,30,34]. Colour measurements are also influenced by sample thickness, background colour behind a sample, the pressure of the colorimeter on the sample, differences in myofibril orientation, and external light [13,35,36]. Finally, many colorimeters, such as the Minolta CR400 and the Nix Pro, require that the sample covers the entire aperture, hence the aperture size becomes important relative to the surface area to be measured. Hence, to accurately measure a large surface area of seafood with heterogenous colour, such as raw salmon, multiple measurements across the surface are required in order to obtain a representative average [37]. However, machine vision systems use a camera and can thus have the whole sample in the field of view, from which a computer extracts the colour information of the whole sample, thus reducing variation between repeat measurements [38].

Colour measuring devices also differ in their ease of use, initial costs, and operating costs. Machine vision system requires a standard lighting system, whereas the Nix and Minolta colorimeters supply their own light and mostly prevent outside light-interference [13,39]. Machine vision systems also require a camera and the computer processing equipment which can vary in cost (~AUD 1000–5000), while the Minolta CR400 costs ~AUD 10,000 and the Nix costs ~AUD 600. The machine vision system is not presently automated, but assuming that sample positioning and image processing can be performed automatically by the machine vision system, the limitation would be the provision of software on a computer to automatically process the images. Measurement with a colorimeter usually requires a human operator to place the device on the sample, and this process has been conducted online, with success, with the Minolta chromameter. The speed of colour measurement depends on the number of repeat measurements needed per sample, but each measurement with each device takes approximately 2–5 s. The ease of use, speed, and price differences influence which device is the most appropriate for each application.

In addition to the type of device used, the optimal number of repeat measurements required will depend on the intended use of the colour data. The acceptable level of difference (ΔE), between the measured colour and the true colour is calculated as the square root of the combined difference between the L*, a*, b* of the measured colour and the true colour, where true colour is defined as the average colour that would be obtained after taking an infinite amount of measurements [11]. Limiting the colour difference to one ΔE is sufficient for sorting meat into quality grades, since one ΔE is the limit for humans when it comes to detecting a colour difference [40,41]. Where the intended use of the colour data is for indications of microbial load, or other shelf- life traits, then a smaller ΔE could be required [42]. Also, differences between treatment groups may not be detected if the variation within a specific group is too high, and using a low number of repeated measurements could result in non-representative average data for the sample. 

This study aims to identify the number of repeat measurements required to reduce the standard error of the mean to an acceptable level, as previously demonstrated by Holman et al. [43] in an experiment involving beef. We have found no published validation data for the number of required repeat colour measurements for Atlantic salmon (*Salmo salar*), banana prawns (*Penaeus merguiensis*), and rockling (*Genypterus tigerinus*), for the three instruments evaluated in this study. Hence, the aim of this study is to determine the number of repeat measurements required to obtain a representative average of the colour of three species of seafood in both raw and cooked state. 

## 2. Materials and Methods

### 2.1. Sample Preparation

Fresh Atlantic salmon (*Salmo salar*) fillets (*n* = 8), banana prawns (*Penaeus merguiensis*) (*n* = 105), and rockling (*Genypterus tigerinus*) fillets (*n* = 8) were purchased from the local market and transported to the laboratory. For the salmon and rockling, the skin was removed and the fillets were sliced to 1 cm thick, then cut into 5 cm × 8 cm samples. The prawns were deveined and their tails were removed. All the samples were left to equilibrate to room temperature (~20 °C) for about 45 min prior to colour measurement, as some foods are known to be thermochromic [44]. The colour measurements described below were obtained from both raw and cooked samples.

### 2.2. Treatments for Calculation of Required Number of Repeat Measurements

Within each species, and for both raw and cooked samples, a factorial experimental design was used, comprising three measuring devices (Nix, Minolta, and Machine vision). In the case of salmon, there were five thickness treatments for raw salmon (fillets stacked to provide 1, 2, 3, 4, and 5 cm thickness) and three thickness treatments for cooked salmon (fillets stacked to provide 1, 3, and 5 cm thickness). In the case of rockling and prawn, a 1 cm sample thickness for both raw and cooked samples was used. For each species, two tile colours were used under each sample: Manhattan black tile (Product code 97681) or white gloss tile (Product code 1000529) (Beaumont Tiles, Melbourne, Australia). For raw and cooked salmon, the order of measuring colour for each thickness treatment was randomised using the Excel (version 2305, Microsoft Inc., Redmond, WA, USA) random number generator, then within the thickness treatment, the order of measurement for the devices and background colour was also randomised. Fifteen repeat measurements were taken with each device for each thickness, each background colour, and each state, and the sample was rotated 25 degrees on a rotating plate between measurements. For rockling and prawns, the order of measurement for the devices and background colour was randomised using the Excel random number generator. Note that the effects of sample thickness and tile colour are not included in this paper, as the focus is on the number of repeat measurements required. A separate forthcoming paper will cover their effects.

### 2.3. Treatments for Calculation of Heterogeneity

In the case of heterogeneity assessment, one photo was taken for all species in each state (raw and cooked) on the white tile at 5 cm thickness, where 1 cm samples were stacked to a depth of 5 cm. Only one photo of each species in each state was used for heterogeneity calculations because the results from the current paper showed that the variation between replicate photos was very low and would not change the heterogeneity calculations.

### 2.4. Cooking Conditions

The salmon and rockling were cooked in a plastic pouch in 80 °C water for 6.25 min to an internal temperature of 70 °C, as recommended by AOAC [45]. Prawns were cooked in boiling salted water (0.36 M NaCl), with a prawn to water ratio of 1:10 (*w*/*v*) for 4 min to reach an internal temperature of 84 °C.

### 2.5. Measuring Devices

For Nix (Nix Pro Color Sensor™, Nix Sensor Ltd., Burlington, ON, Canada) and Minolta (Minolta CR400 colorimeter, Konica–Minolta, Tokyo, Japan), the measuring head was moved between measurements to obtain representative measurements of the entire sample surface. The Minolta CR400, with an 8 mm aperture diameter, was set to D65 illuminant, 2° observer, and the Nix, with a 10 mm aperture diameter, was set to D65 illuminant and a 10° observer angle, as specified by ISO/CIE [46]. All measurements were taken with the sample placed in a light box (Photobench 120, Ortery, Irvine, CA, USA), which prevented light interference from the surrounding room. The Minolta was calibrated daily with a white tile supplied with the Minolta (Y = 84.9, X = 0.3195, y = 0.3368). The Nix was calibrated in the factory, and no further calibration was possible. All results are expressed as CIE L*, a*, and b*.

The Canon camera (EOS 7D DSLR camera, Canon, Tokyo, Japan) was set to manual mode and an ISO speed of 400, an aperture of f/5.6, an exposure time of 1/60 s, and a 35 mm focal length. This resulted in neutral exposure to an 18% middle grey card, as recommended by photography professionals [47]. The lens barrel was placed at the top of the light box, which prevented any light interference, as shown in Supplementary Figure S1. The lights used were D65 LEDs (Flex Mk2 2–meter LED strip, MediaLight, Melbourne, VIC, Australia) adhered manually to the light box ceiling in a square, with 310 lux at the surface of the sample. The samples were positioned so that the angle between the illuminant and the camera was 45°, as diffuse reflection, which is responsible for colour, occurs at this angle [48] (as cited in [49]). 

Pictures of each colour measurement device are shown in Supplementary Figure S2.

### 2.6. Photo Processing

The digital photos obtained were raw files (CR2) and were processed in Lightroom version 6.2 (Adobe, San Jose, CA, USA) using a colour calibration card (X–rite mini, X–rite Inc., Grand Rapids, MI, USA) to adjust for the camera and illumination temperature (D65) and were then saved as a .TIFF file. These photos were cropped in Photoshop version 23 (Adobe, San Jose, CA, USA) to isolate the sample, and also to remove any artefacts. These photos were then saved as a .png file with no compression. The average colour in the red, green, and blue (RGB) colour space was calculated for each digital photo and converted into CIE L*, a*, b* colour space values.

### 2.7. Statistical Analysis

The number of repeat measurements (*r*) required was determined using two methods.

#### 2.7.1. Repeat Measurement Determination

Method 1—In the standard error method, the number of required repeat measurements was defined as when the change in standard error between *r* and *r* + 1 reached <1%, as described by Holman, Diffey, Logan, Mortimer, and Hopkins [34]. To calculate the standard error, Genstat (VSN International Ltd., Hemel Hempstead, England, UK, version 18.2.0.18409) was used to run a Linear Mixed Model analysis separately for each species (salmon, rockling, and prawns), cooking state (raw, cooked), and colour parameter (L*, a*, b*). For salmon, the data included all repeat measurements, and used thickness, tile colour, and their interaction in the fixed model, and the random model contained the sample number. This model structure was used for completeness, but the effects of thickness and tile colour are not reported here, which is similar to the approach used by Holman et al. [43]. For rockling and prawns, the data included all repeat measurements, and used tile colour in the fixed model, and the random model contained the sample number. The sample and residual variance were obtained for each model and compared using a linear mixed model to quantitate differences across colour parameter, species, and device. The standard error was calculated according to Equation (1) for values of *r* until the change in standard error between *r* and *r* + 1 was <1%. As three calculations were conducted (L*, a*, and b*, for each sample), the required number of repeat measurements was defined as the highest value obtained across L*, a*, b*. Devices were compared using one-way ANOVA in Minitab (version 19.1.1) to determine the significant differences between number of required repeat measurements.

(1) $$Standard\;error=\surd (Sample\;Variance+\frac{Residual\;Variance}{r}),$$

Method 2—In the total colour difference (ΔE) method, the number of required repeat measurements was defined as when the colour difference (ΔE) between the average colour at each value of *r* and *r* = 15 was <1 and never increased above 1 for all greater values of *r*. Instead of using the traditional method for calculating ∆E (∆E = √[(*L*_1_* − *L*_2_*)^2^ + (*a*_1_* − *a*_2_*)^2^ + (*b*_1_* − *b*_2_*)^2^]) used by Oliveira and Balaban [37] and others, the CIEDE2000 (ΔE_00_) was calculated as given in Luo et al. [50], which creates values that are close to the human perception of colour difference [51]. The calculation was performed in Microsoft Excel (version 2305) using an add-in developed by Garcia [52].

The order of the 15 repeat measurements for each sample was randomised, and the raw average was calculated sequentially over the range of *r* from 1 to 15. The colour difference was calculated between the average at each value of *r* compared to the final average at *r* = 15. The required *r* was the lowest number for which the colour difference was below 1 and stayed below 1 for all greater values of *r*. Randomisation and calculation steps were undertaken 12 times to reduce the effects of variation in simulated measurement order. Hence, a value for *r* was obtained where, in 95% of cases, further repeat measurements did not give rise to a colour difference of more than 1.

#### 2.7.2. Heterogeneity

Two methods were used to estimate the colour heterogeneity of the sample using the photo images. 

Method 1—This was based on the definition of colour uniformity from Oliveira and Balaban [37]: heterogeneity is defined as the average standard deviation of L*, a*, b* values from all pixels in the sample. 

Method 2—In order to simulate measurement with a Minolta colorimeter, 15 circles of the same size as the Minolta aperture were selected on the photos to acquire 15 L*, a*, b* values. The 15 simulated Minolta L*, a*, b* measurements were averaged, then the colour difference was calculated for each simulated measurement compared to the average colour. These colour difference values were then averaged.

For each method, an ANOVA was run in Minitab (version 19.1.1) to determine significant differences between samples.

## 3. Results

Table 1 shows the average colour measured for each species in each state for each device. Overall, the standard deviation for Nix and Minolta colour values was lower than for machine vision.

### 3.1. Method 1

**Figure 1:**
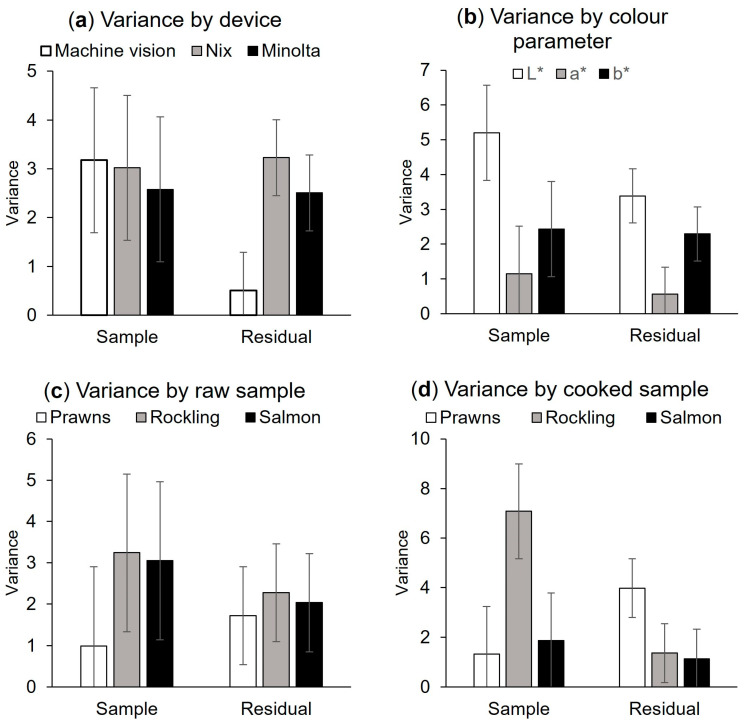
Effect of: (**a**) device for all (cooked and raw) samples (sample variance, *p* = 0.917; residual variance, *p* = 0.003); (**b**) colour parameter for all (cooked and raw) samples (sample variance, *p* = 0.015); residual variance, *p* = 0.003); (**c**) species for raw samples (sample variance, *p* = 0.031; residual variance, *p* = 0.216), (**d**) species for cooked samples (sample variance, *p* = 0.031; residual variance, *p* = 0.216), on the residual and sample variance for prawns (*Penaeus merguiensis*), rockling (*Genypterus tigerinus*), and salmon (*Salmo salar*). The vertical lines represent the least significant difference (LSD), which only applies to comparisons within a variance type (sample or residual).

The sample variance and residual variance for each device, colour parameter, and seafood species are shown in Figure 1.

Machine vision had a much lower residual variance than the two colorimeters, indicating there was less difference between repeat measurements (Figure 1). Whilst there were no differences between devices for sample variance, the standard deviation for machine vision was higher than for the Minolta: 1.80 compared to 1.43, respectively. L* tended to have high variance, whereas a* had low variance, and cooked rockling had the highest sample variance.

**Figure 2:**
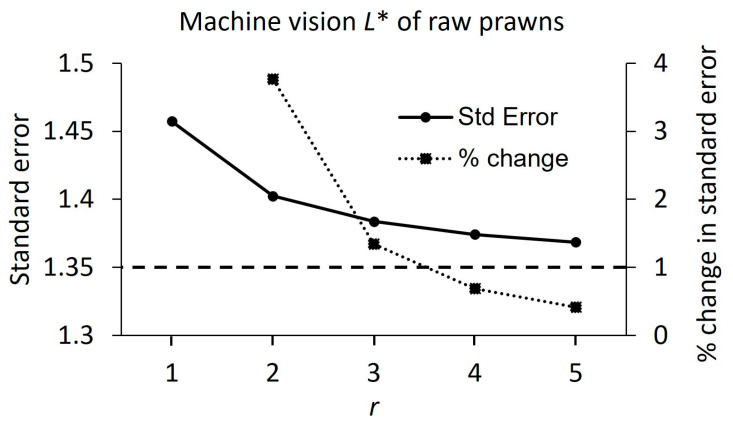
The standard error (left y-axis) and the change in standard error (%) (right y-axis) vs. number of repeat measurements (*r*) for the machine vision measurements of L* for raw prawns (*Penaeus merguiensis*). A reference line is drawn at percent change in standard error = 1.0, and below this line are values of *r* which are higher than required.

The required repeat measurements, using Method 1, for each species in the raw and cooked state and using each device, are shown individually for L*, a*, and b*, and overall, in Table 2. To illustrate how these were calculated, Figure 2 shows the standard error and percent change in standard error plotted against the number of repeat measurements (*r*), for L* measured using machine vision on raw prawns, as an example. In this case, the required *r* would be three, because the reduction in standard error achieved by using one more repeat measurement was lower than 1% of the standard error at *r* = 3.

To reduce the change in standard error to below 1%, machine vision required fewer repeat measurements in comparison to Nix and Minolta (*p* < 0.05 for both), whereas Nix and Minolta did not differ (*p* > 0.05). In contrast, in three samples out of the six, machine vision had higher standard error when it was modelled with *r* = 15, as shown in Supplementary Figure S3. The higher standard error was caused by a higher sample variance for machine vision, as in all but one sample, the residual variance was lower.

### 3.2. Method 2

**Figure 3:**
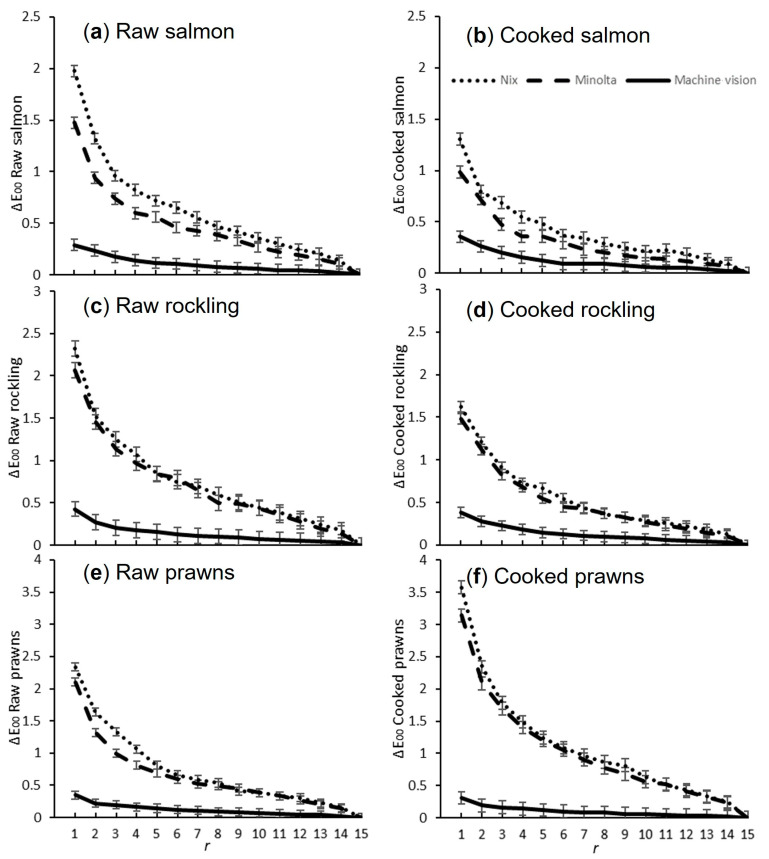
Average ΔE_00_ (colour difference) between the final average colour and the average colour at each of the 15 repeat measurements (*r*) for (**a**) raw salmon (*Salmo salar*), (**b**) cooked salmon, (**c**) raw rockling (*Genypterus tigerinus*), (**d**) cooked rockling, (**e**) raw prawn (*Penaeus merguiensis*), and (**f**) cooked prawn, measured with each device. Vertical lines represent the least significant difference (LSD). *p* < 0.001 in each graph for effect of device and *r*.

Figure 3 shows the average colour difference (ΔE_00_) between the current average and the final average colour for each sample as *r* increased, clearly showing that the colour difference for machine vision was lower than for both colorimeters. For all samples and all devices, as the number of measurements increased, the average colour difference was equivalent to the final average at *r* = 15.

The number of repeat measurements required for machine vision was 1, while the colorimeters required an average of 10.5 (Table 2), using the metric that in 95% of calculations, the colour difference was less than one.

### 3.3. Heterogeneity Estimations

**Figure 4:**
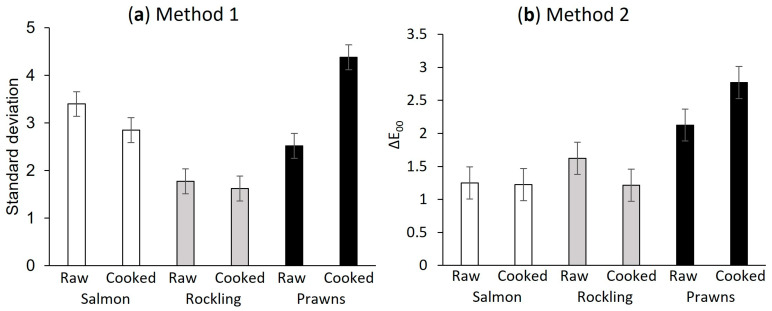
Colour heterogeneity across the sample measured with (**a**) Method 1, literature method based on all pixels within the sample, and (**b**) Method 2, average CIEDE2000 (∆E_00_) using simulated Minolta readings. Vertical lines represent the least significant difference (LSD). Method 1, *p* < 0.001; Method 2, *p* < 0.001.

In order to understand variation in the number of repeat measurements needed between species, the standard deviation was calculated as a measure of intra-sample colour heterogeneity and is shown in Figure 4. For Method 1, there were differences in the standard deviation between samples *(*p* <* 0.001), with cooked prawns having the highest standard deviation (heterogeneity). For Method 2, using colour difference (∆E_00_) as the metric, again, cooked prawns had the highest heterogeneity, but in contrast to the first method, raw and cooked salmon did not have high heterogeneity.

## 4. Discussion

The most important result was that machine vision generally required fewer repeat measurements than the Nix and Minolta colorimeters. The colorimeters required more repeat measurements due to the higher variability between repeat measurements. This is evident from the higher residual variance and higher colour difference for most values of *r* for Nix and Minolta, indicating that they are less precise than machine vision. Although the Nix had a 56% bigger aperture compared to the Minolta (78.5 mm^2^, 50.3 mm^2^, respectively), the Nix required a similar number of repeat measurements to the Minolta using calculations of both Method 1 and 2. Holman, Collins, Kilgannon, and Hopkins [43] proposed that the smaller aperture of the Minolta could be used to avoid heterogenous areas (such as the fat stripes in raw salmon) and that this would reduce the variability of the repeated measurements and reduce how many are required to representatively capture the colour of the sample. However, colorimeter results may also be impacted by edge loss, which is defined as the loss of illuminant from the sample if it scatters outside the area of the aperture [53]. There may also have been some variability in the colorimeters due to pressing the sample too hard, or due to small gaps between the sample surface and the detector, allowing light in and out [35]. Sample heterogeneity, and possibly variations in illumination and pressure, likely contributed to the colorimeters requiring more repeat measurements.

Whilst samples with high heterogeneity tended to require more repeat measurements, there were exceptions. When using Method 1, cooked prawns required a similar number of repeat measurements to raw prawns, even though the residual variance of cooked prawns was higher than that of raw prawns. This discrepancy is most likely because the calculation used to determine the number of repeated measurements required includes the sample variance, which was also higher in cooked prawns. In addition, although raw rockling had a lower heterogeneity, based on the average colour difference (Method 2) between the simulated repeat measurements, than raw prawns, it required more repeat measurements with the colorimeters. Overall, the differences between the number of repeat measurements required between samples were mostly explained by the colour heterogeneity.

This study has highlighted the importance of validating seafood colour measurement techniques rather than adopting published protocols from other species, particularly as there is very little literature on instrumental colour measurement of seafood species. Across the species, cooked/raw and colour parameters, the Nix colorimeter required a range of repeat measurements, *r,* of 22, 19, 17, 5, 8, and 9, which was quite different to the recommended *r* of 7, 4, and 3 for beef, lamb, and beef, respectively [34,43,54]. However, the recommendations of seven and four for beef and lamb are low because only the a* value needs to be sufficiently precise to correlate with the consumer acceptance threshold, not L* or b* [34,43,55,56]. If L* and b* were required to be as precise as a*, then 10 repeat measurements would be needed for aged beef and 9 for lamb [34,43]. For raw salmon, chroma ([a*^2^ + b*^2^]^1/2^) is correlated to visual colour assessment, and since a* was the limiting factor for raw salmon repeat measurement, focusing on the relevant colour parameter would not reduce the requirement [57].

For the Minolta, the repeat measurements required for salmon (6 and 10), prawns (23 and 15), and rockling (15 and 3) were much higher than the triplicate measurements that are recommended for precise determination of the colour of red meat [11,58]. Oliveira and Balaban [37] reported that three repeat measurements on sturgeon fillets obtained using the Minolta CR–200 were inadequate. This was based on a machine vision system having higher standard deviations of L*, a*, and b* values between replicate sturgeon fillets than the Minolta, indicating a diversity of colour that was not captured by the Minolta.

Based on our study, we would recommend an *r* for machine vision seven for raw salmon, and three for cooked salmon, raw rockling, cooked rockling, raw prawns, and cooked prawns. These results align with the study of Milovanovic et al. [59], who recommended that three replicate measurements are required for machine vision systems on dairy products, in agreement with previous recommendations for colorimeters. With machine vision, three repeat measurements were acceptable for all species except raw salmon, using the standard error minimisation method (Method 1). However, using Method 2, only a single repeat measurement was needed for each sample. In summary, the colorimeters generally required more repeat measurements than recommended in the literature, and machine vision required the same or fewer repeat measurements than stated in the literature.

### Limitations

There are two flaws that limit the reliability of the calculation of the number of repeat measurements required when using Method 2. First, this method requires an infinite number of replicates to obtain the true colour of the sample against which the colour difference is calculated at each *r*. Secondly, the data set used to create the final average was the same data set used to calculate the cumulative average at each *r*. Thus, as *r* approached 15, the colour difference fell to 0. If fewer than fifteen repeat measurements were used to create the comparison data, the range of possible required *r* would shrink, thus lowering the average.

## 5. Conclusions

The number of repeat measurements required varies by sample type and is related to colour heterogeneity. The number of repeat measurements required was higher than recommended in the literature for colorimeters when measuring meat. For machine vision, the number of recommended repeat measurements was generally similar to, or lower than, that described in the literature on dairy products.

## Figures and Tables

**Table 1:** Average L*, a* and b* values for raw and cooked banana prawn (*Penaeus merguiensis*), rockling (*Genypterus tigerinus*), and Atlantic salmon (*Salmo salar*), measured by Nix, Minolta, and machine vision ^1^. Standard deviation in brackets.

	N	Nix			Minolta			Machine Vision		
		L*	a*	b*	L*	a*	b*	L*	a*	b*
Raw prawns	7 ^2^	46.44 (2.48)	−1.72 (0.71)	−5.91 (2.71)	45.54 (1.99)	−1.14 (0.66)	−4.93 (2.55)	47.67 (6.88)	1.10 (1.96)	11.24 (5.73)
Cooked prawns	7	66.96 (2.82)	−3.06 (1.56)	3.92 (3.42)	65.50 (2.66)	−2.48 (1.42)	5.78 (3.39)	55.95 (3.81)	3.32 (2.07)	19.98 (3.13)
Raw rockling	8	53.37 (3.26)	−1.57 (1.00)	−5.23 (2.20)	52.77 (2.89)	−1.06 (0.92)	−3.82 (2.18)	53.10 (6.73)	2.76 (2.99)	6.92 (4.03)
Cooked rockling	8	79.87 (5.21)	−2.92 (0.81)	2.24 (2.07)	80.05 (4.71)	−2.01 (0.77)	3.00 (1.90)	61.12 (3.07)	0.80 (0.94)	8.40 (1.45)
Raw salmon	8	39.65 (3.27)	13.45 (1.76)	15.54 (2.23)	41.10 (2.72)	11.38 (1.44)	15.14 (2.05)	32.95 (2.75)	32.02 (3.10)	38.51 (4.08)
Cooked salmon	8	78.33 (2.41)	14.80 (1.37)	19.33 (1.52)	76.76 (1.66)	15.00 (1.24)	21.26 (1.65)	54.19 (1.59)	18.38 (1.42)	22.42 (2.05)

^1^ Please refer to the Section 2 for instrument details. ^2^ Each replicate of raw prawns and cooked prawns required several prawns, hence the total number of prawns used was *n* = 105.

**Table 2:** Required repeated measurements (*r*) for Method 1 and 2 for raw and cooked prawn (*Penaeus merguiensis*), rockling (*Genypterus tigerinus*), and salmon (*Salmo salar*).

		Method 1				Method 2
Sample	Device	L*	a*	b*	Highest	
Raw prawns	Nix	22	8	9	22	9
	Minolta	23	8	10	23	9
	Machine vision	3	2	2	3	1
Cooked prawns	Nix	10	10	19	19	12
	Minolta	10	10	15	15	11
	Machine vision	3	2	3	3	1
Raw rockling	Nix	17	4	4	17	11
	Minolta	15	5	4	15	11
	Machine vision	3	2	3	3	1
Cooked rockling	Nix	3	5	3	5	8
	Minolta	3	3	3	3	8
	Machine vision	1	3	3	3	1
Raw salmon	Nix	6	8	7	8	9
	Minolta	5	6	6	6	7
	Machine vision	3	7	5	7	1
Cooked salmon	Nix	9	6	5	9	7
	Minolta	10	6	4	10	5
	Machine vision	3	2	2	3	1

## Data Availability

The original contributions presented in the study are included in the article/Supplementary Materials, further inquiries can be directed to the corresponding author.

## Supplementary Materials

The following supporting information can be downloaded at: https://www.mdpi.com/article/10.3390/foods13071110/s1, Figure S1: Lightbox setup for photography and colorimeter measurement (please refer to online version for Figure with colours). Figure S2: Colour measurement devices - Canon EOS 7D DSLR camera (left), Nix and associated smartphone app (middle), and Minolta CR400 (right); Figure S3: Calculated standard error at *r* = 15 using Equation (1).
