# Supplementary material for: Machine Vision Requires Fewer Repeat Measurements than Colorimeters for Precise Seafood Colour Measurement
Source: Foods. 2024 Apr 4;13(7):1110. doi: 10.3390/foods13071110 (PMC11011751; doi:10.3390/foods13071110)
Supplement: Supplementary file 1 [file foods-13-01110-s001.zip › foods-2925454-supplementary.pdf]

foods

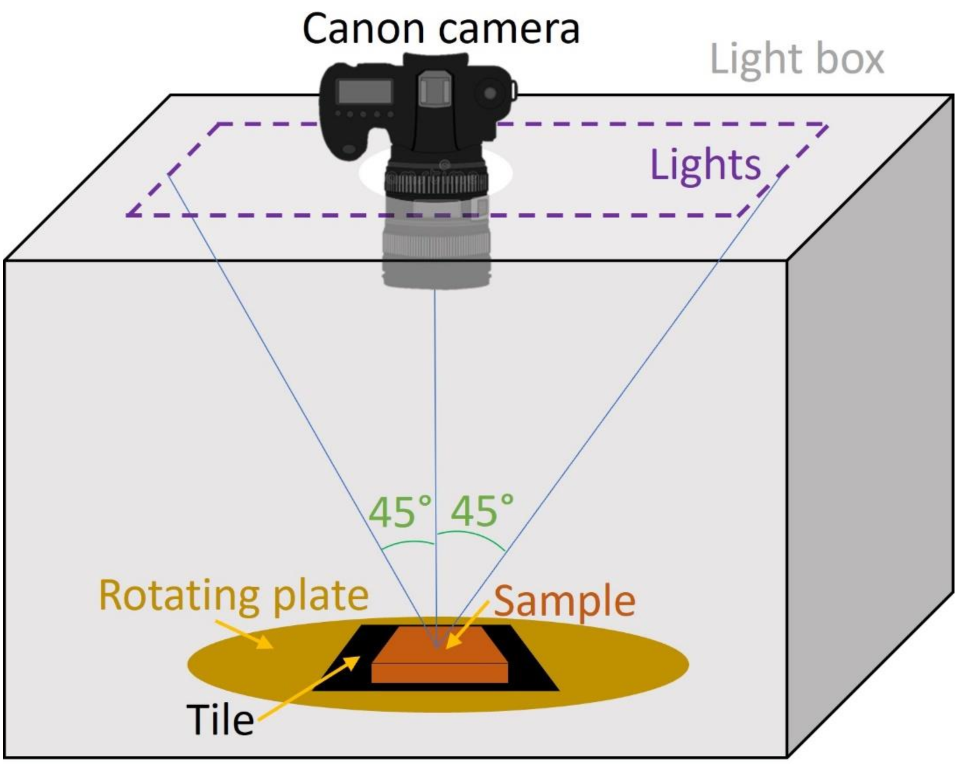

Figure S1. Lightbox setup for photography and colorimeter measurement (please refer to online version for Figure with colours).

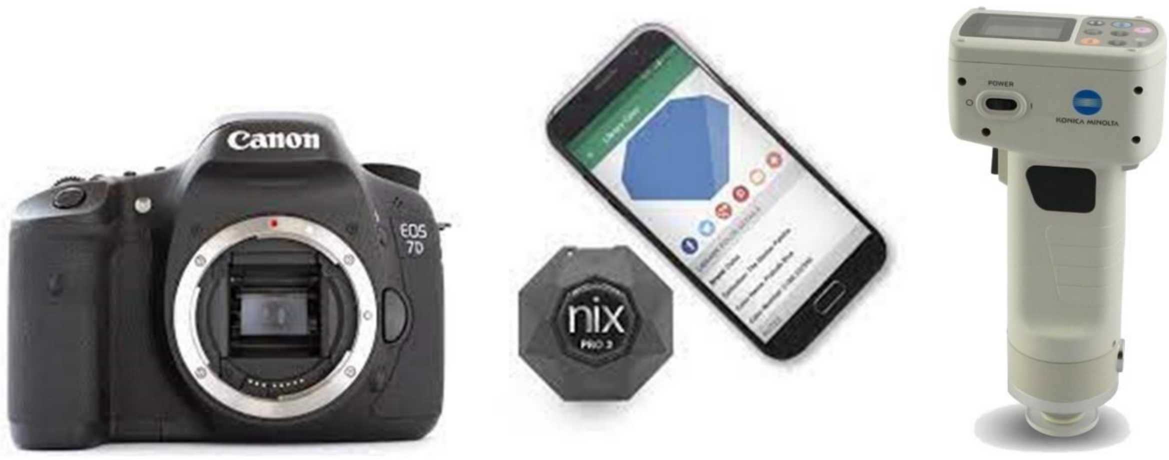

Figure S2. Colour measurement devices; Canon EOS 7D DSLR camera (left), Nix and associated smartphone app (middle), and Minolta CR400 (right).

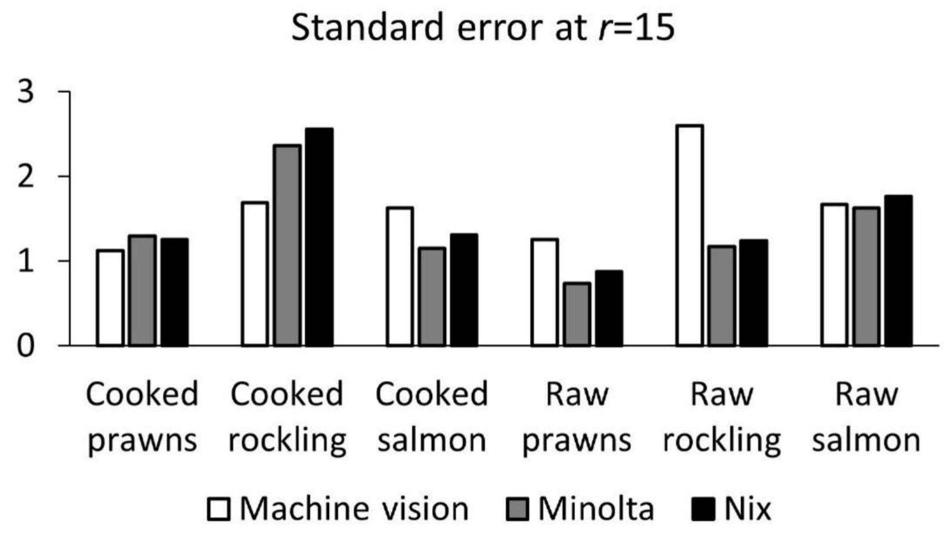

Figure S3. Calculated standard error at *r*=15 using equation 1.
